# Adverse drug reactions and drug interactions in the treatment of hospitalized patients with coronavirus disease 2019 (COVID-19)

**DOI:** 10.1017/ash.2021.196

**Published:** 2021-10-28

**Authors:** Tatiana A. Marins, Alexandre R. Marra, Michael B. Edmond, Ligia Regina Prystaj Colombo, Sthephanie Favalli Vieira, Fernanda de Oliveira Xavier, Alessandra Gomes Chauvin, João Renato Rebello Pinho, Silvana M. de Almeida, Marcelino Souza Durão Junior

**Affiliations:** 1 Department of Clinical Pharmacy, Hospital Israelita Albert Einstein, São Paulo, Brazil; 2 Instituto Israelita de Ensino e Pesquisa Albert Einstein, Hospital Israelita Albert Einstein, São Paulo, Brazil; 3 Carver College of Medicine, University of Iowa, Iowa City, Iowa, United States; 4 West Virginia University School of Medicine, Morgantown, West Virginia, United States; 5 Special Techniques Laboratory, Hospital Israelita Albert Einstein, São Paulo, Brazil; 6 Division of Medical Practice, Hospital Israelita Albert Einstein, São Paulo, Brazil

**Keywords:** adverse drug reactions, drug interactions, COVID-19, off-label

## Abstract

**Objectives:**

To identify drugs that were administered off label to hospitalized patients with suspected coronavirus disease 2019 (COVID-19) and to identify adverse drug reactions (ADRs) and drug–drug interactions associated with these therapies.

**Methods:**

This case–control study was conducted in a Brazilian hospital from March to April 2020 among patients with suspected COVID-19, comparing those with positive severe acute respiratory coronavirus virus 2 (SARS-CoV-2) reverse-transcriptase polymerase chain reaction (RT-PCR) results and those with negative results.

**Results:**

The most commonly used medications in both groups were azithromycin and hydroxychloroquine. There was a significantly higher prevalence of reactions among patients with positive RT-PCR for SARS-CoV-2 (48.5% vs 28.8%; *P* = .008) in the propensity score–matched cohort, and the most commonly reported ADRs among these patients were diarrhea (43.8%), elevated liver enzymes (31.3%), and nausea and vomiting (29.7%).

**Conclusions:**

Our data demonstrate that ADRs and drug–drug interactions are common with off-label treatments for COVID-19.

In early January 2020, a new type of coronavirus, severe acute respiratory coronavirus virus 2 (SARS-CoV-2), was identified by the Chinese as being the agent responsible for causing the coronavirus disease 2019 (COVID-19), which primarily affects the respiratory tract.^1–4^ After the World Health Organization (WHO) declared the outbreak a pandemic, a quest for efficacious treatments began, and several drugs were used in off-label regimens. Several clinical studies were initiated in different countries aimed at finding vaccines and specific treatments for COVID-19.

In the beginning of the pandemic, in vitro studies showed promise for some drugs.^5,6^ Several pharmacological therapies were considered, including hydroxychloroquine or chloroquine, with or without azithromycin, tocilizumab, and lopinavir/ritonavir.^7,8^ These drugs started to be prescribed off label worldwide. Subsequently, randomized clinical trials in humans started to demonstrate the safety and efficacy of these therapies.^7^ In this study, we aimed to identify drugs that were administered in off-label regimens to hospitalized patients with suspected COVID-19, to identify adverse drug reactions (ADRs) and prevalent drug–drug interactions in these therapies, and to evaluate risk factors for ADRs to the drugs administered to patients suspected of having COVID-19 and those with confirmed COVID-19.

## Methods

This study was conducted in a private, tertiary-care hospital focused on high-complexity treatments, including a transplant program, located in São Paulo, Brazil. This case–control study included hospitalized patients suspected of COVID-19 via a retrospective analysis of medical records from March 1 through April 30, 2020. Patients were identified through a report extracted from the hospital data management system and through a logistics system that identified the use of drugs deemed off label. The study was approved by the institution’s research ethics committee and informed consent was not required.

The case group comprised patients with a positive reverse-transcriptase polymerase chain reaction test (RT-PCR) for SARS-CoV-2, These cases were matched with the control group composed of patients with negative RT-PCR tests for SARS-CoV-2. The diagnostic confirmation for COVID-19 was performed using RT-PCR on specimens obtained via nasopharyngeal or oropharyngeal swab, bronchoalveolar lavage, or secretions collected via tracheostomy, according to the protocol instituted at the hospital. The technique of amplifying nucleic acids for detection of SARS-CoV-2 RNA adopted by our laboratory during the study period followed the methodology developed by the Charité – Universitätsmedizin Berlin Institute of Virology and the German Centre for Infection Research, both located in Berlin, Germany.

When analyzing the medical histories of patients hospitalized and suspected of COVID-19 infection, patients aged <18 years and outpatients were excluded. Data collection was performed according to a standard questionnaire form via REDCap.^9,10^ It included the following variables: age, sex, body mass index (BMI), date of admission, period of hospitalization, type of inpatient unit, severity according to the Simplified Acute Physiology Score III (SAPS-III) scale, drugs prescribed during COVID-19 treatment, treatment duration, dose, frequency, and route of administration.

Therapies considered off label for treating COVID-19 include the following drugs: hydroxychloroquine sulfate, azithromycin, tocilizumab, lopinavir/ritonavir, and interferon. The use of antibacterial agents for pneumonia treatment was considered a supplemental therapy. The following standard of care was used at our institution during the study period: After identifying the suspected patient with acute respiratory symptoms with or without fever, the RT-PCR for SARS-CoV-2 and the molecular panel of respiratory pathogens were collected. The physician could start oseltamivir until the molecular panel results were received, suspend it after obtaining a negative test result, and administer antibiotics for the treatment of pneumonia. At the time, no randomized clinical trials had proven the clear benefit of these drugs for the treatment of COVID-19 and in vitro studies suggested possible efficacy, considering the severity of illness.

Comorbid conditions of the study patients were identified, as well as baseline assays for potassium, magnesium, and ionic calcium to identify the presence of electrolyte disturbances. The primary outcome considered in our study was ADRs, and the secondary outcomes were the presence of drug interactions and/or drugs that have potential risks for QT interval prolongation.

The ADRs were identified in a thorough reading of medical and nursing evaluations in the medical records of the patient’s hospital stay. This search was conducted by 5 investigators. The patients were divided using the Naranjo algorithm, a tool used to classify ADRs into 5 probability categories: defined, probable, possible, conditional, or doubtful.^11^

To analyze ADRs and drug–drug interactions, we used the Micromedex and Up To Date electronic databases, the package inserts of each drug, and guidance from the Brazilian Health Surveillance Agency (Portuguese acronym ANVISA), the Brazilian Ministry of Health, and the World Health Organization (WHO). The Credible Meds website (http://www.crediblemeds.org), created by a nonprofit organization, was also used to evaluate drugs that have potential risks for QT interval prolongation.^12^ This database classifies the risk for QT interval prolongation and the induction of Torsades de Pointes (TdP) as known risk, possible risk, or conditional risk.^12^ Drug–drug interactions were classified as contraindicated, high severity, or moderate severity. We also verified whether an electrocardiogram (ECG) had been performed before and during treatment, as in the case of drug–drug interaction related to the prolongation of the QT interval. The QT interval was considered prolonged if the corrected QT (QTc) was >470 ms for men and >480 ms for women.^13^

### Statistical analysis

The data were characterized using means, standard deviations, minimum and maximum values, medians, and interquartile intervals (for the quantitative variables) as well as absolute and relative frequencies (for the qualitative variables).^14^

Comparisons between groups were analyzed using the χ^2^ or Fisher exact test for categorical variables and the *t* test and Mann-Whitney *U* test for continuous variables, depending on the distribution of data. Data normality was assessed using the Shapiro-Wilk test, box-plot graphs, histograms, and quantile comparison graphs.^14^ Due to the differences in proportions between PCR-positive and PCR-negative groups, 2:1 matching was carried out using a propensity score. The matching method was optimal pair matching,^15^ and the propensity score was performed based on a logistic regression model. We considered the following as covariables: age, body mass index, sex, type of inpatient unit, and underlying diseases (heart diseases, lung diseases, and diabetes).^16^

After matching, a model of generalized estimation equations was proposed to estimate the chance of ADRs between the groups, considering the dependence between cases and controls.^17^ The drugs analyzed for the treatment of COVID-19 were limited to hydroxychloroquine and azithromycin because the other drugs were used infrequently. Treatment duration, drug interaction, length of hospitalization period, and drugs at risk for QT interval prolongation were considered covariables.

The analyses were performed using the Statistical Package for the Social Sciences software (SPSS) R integration package version 26.0.^18^ The significance level was set at 0.05.^19^

## Results

The full cohort comprised 254 patients with suspected COVID-19. Due to the differences in proportion between SARS-CoV-2–positive and –negative groups, 2:1 matching was carried out using a propensity score, resulting in 66 pairs and 198 patients. The propensity model had an area under the curve (AUC) of 0.619. Table 1 shows the characteristics of each group.

**Table 1.** Characteristics of Inpatients

Variable	Full Cohort				*P* Value	Propensity Score–Matched Cohort				*P* Value
	Negative SARS-CoV-2 RT-PCR (n = 66)		Positive SARS-CoV-2 RT-PCR (n = 188)			Negative SARS-CoV-2 RT-PCR (n = 66)		Positive SARS-CoV-2 RT-PCR (n = 32)		
	No.	%^a^	No.	%^a^		No.	%^a^	No.	%^a^	
Sex, male	35	53.0	116	61.7	.217^b^	35	53.0	68	51.5	.841^b^
Age, mean y, SD	65.1	16.4	63.8	16.9	.578^c^	65.1	16.4	65.7	18.0	.831^c^
Weight mean kg, SD	74.78	16.17	80.56	18.50	.025^c^	74.8	16.2	75.7	16.8	.724^c^
Height, mean cm, SD	167.4	10.37	170.4	10.1	.041^c^	167.4	10.4	169.0	10.2	.326^c^
Body mass index	26.5	4.2	27.5	4.8	.119^c^	26.5	4.2	26.3	4.7	.844^c^
**Level of care**					.664^b^					.912^b^
Ward	47	71.2	125	66.5		47	71.2	93	70.5	
ICU or step-down unit	19	28.8	63	33.5		19	28.8	39	29.5	
**SAPSIII ICU admission score**					.938^c^					.504^c^
Mean, SD	58.0	15.6	58.4	12.4		58.0	15.6	61.8	11.2	
In-hospital mortality	6	9.1	20	10.6	.721^b^	6	9.1	16	12.1	.522^b^
Length of stay, median d, IQR	7.0	(4–13)	11.0	(5–22)	.004^d^	7.0	(4–13)	11.0	(6–31)	.001^d^
Coexisting condition	57	86.4	145	77.1%	.110^b^	57	86.4	113	85.6	.885^b^
Lung disease	8	14.0	20	13.8	.964^b^	8	12.1	16	12.1	.989^b^
Heart disease	32	56.1	88	60.7	.553^b^	32	48.5	65	49.2	.975^b^
Kidney disease	5	8.8	9	6.2	.518_b_	5	8.8	7	6.2	.536^b^
Liver disease	1	1.8	2	1.4	>.999^b^	1	1.8	1	0.9	>.999^b^
Diabetes	10	17.5	48	33.1	.028^b^	10	15.2	20	15.2	.989^b^
Nervous system disease	10	17.5	27	18.6	.859^b^	10	17.5	23	20.4	.662^b^
Cancer	14	24.6	29	20.0	.476^b^	14	24.6	28	24.8	.975^b^
Thyroid disease	9	15.8	31	21.4	.370^b^	9	15.8	26	23.0	.272^b^
Other coexisting conditions	13	22.8	37	25.5	.688^b^	13	22.8	29	25.7	.683^b^
Use of antimicrobial	62	93.9	176	93.6	.926^b^	62	93.9	125	94.7	.826^b^

Note. SARS-CoV-2, severe acute respiratory coronavirus virus 2; RT-PCR, reverse transcriptase-polumerase chain reaction; SD, standard deviation; ICU, intensive care unit; SAPSIII, Simplefied Acute Physiology Score III; IQR, interquartile range. Definitions: Lung diseases were respiratory diseases such as asthma and chronic obstructive pulmonary disease; heart diseases were high blood pressure and coronary heart disease; kidney diseases were chronic or acute renal failure; liver diseases were liver failure and hepatitis; nervous system diseases were Alzheimer’s and delirium.

a Percentage unless otherwise specified.

b Data presented as n and % compared using the χ^2^ test.

c Data presented as mean (SD), compared using the Student *t* test.

d Data presented as median and interquartile range, compared by Mann-Whitney test.

Table 2 shows treatment by group of drugs and time of treatment. The most commonly used medication in both groups was azithromycin, which was used in ∼86% of patients. Among the patients in the positive group, 84.8% also received hydroxychloroquine and more than half received concomitant hydroxychloroquine and azithromycin. The frequency of other drugs was low in both groups, and none of the patients received interferon.

**Table 2.** Treatment of COVID-19 by Group

Variable	Full Cohort				*P* Value	Propensity Score–Matched Cohort				*P* Value
	Negative SARS-CoV-2 RT-PCR (n = 66)		Positive SARS-CoV-2 RT-PCR (n = 188)			Negative SARS-CoV-2 RT-PCR (n = 66)		Positive SARS-CoV-2 RT-PCR (n = 132)		
	No.	%^a^	No.	%^a^		No.	%^a^	No.	%^a^	
Hydroxychloroquine	34	51.5	158	84.0	<.001^b^	34	51.5	112	84.8	<.001^b^
Treatment duration, median d (IQR)	2.5	1–5	7.0	4–10	<.001^c^	2.5	1–5	7.0	4–10	<.001^c^
Azithromycin	56	84.8	162	86.2	.791^b^	56	84.8	117	88.6	.449^b^
Treatment duration, median d (IQR)	4.0	2–6	6.0	4–9	<.001^c^	4.0	2–6	6.0	4–10	<.001^c^
Associated and isolated use										
Hydroxychloroquine + Azithromycin	27	42.9	135	73.0		27	42.9	98	74.8	
Azithromycin	29	46.0	27	14.6	<.001^b^	29	46.0	19	14.5	<.001^b^
Hydroxychloroquine	7	11.1	23	12.4		7	11.1	14	10.7	
Lopinavir/Ritonavir	3	4.5	22	11.7	.093^b^	3	4.5	12	9.1	.255^b^
Treatment duration, median d (IQR)	1.0	1–1	6.5	5–9	.008^c^	1.0	1–1	8.0	5.5–9.5	.009^c^
Tocilizumab	0	0.0	14	7.4	.024^d^	0	0.0	10	7.6	.022^d^
Treatment duration, median d (IQR)	…	…	1.0	1–2	…	…	…	1.0	1–1	…

Note. SARS-CoV-2, severe acute respiratory coronavirus virus 2; RT-PCR, reverse transcriptase-polymerase chain reaction; IQR, interquartile range.

a Percentage unless otherwise specified.

b Data presented as no. and %, compared using the χ^2^ test.

c Data presented as median and interquartile range, compared using the Mann-Whitney *U* test.

d Data presented as no. and % compared using the Fisher exact test.

We detected a significantly higher prevalence of ADRs among PCR-positive patients (48.5% vs 28.8%; *P* = .008). The most common reaction among PCR-positive patients who had an ADR was diarrhea (43.8%), followed by nausea and vomiting (29.7%) and elevated liver enzymes (31.3%) (Table 3). Among the patients in the negative PCR group who had ADRs, the most common reaction was also diarrhea (36.8%). Some patients had >1 ADR simultaneously.

**Table 3.** Adverse Drugs Reactions and Drug–Drug Interactions by Group

Variable	Full Cohort				*P* Value	Propensity Score–Matched Cohort				*P* Value
	Negative SARS-CoV-2 RT-PCR (n = 66)		Positive SARS-CoV-2 RT-PCR (n = 188)			Negative SARS-CoV-2 RT-PCR (n = 66)		Positive SARS-CoV-2 RT-PCR (n = 132)		
	No.	%^a^	No.	%^a^		No.	%^a^	No.	%^a^	
Presence of any adverse drugs reaction^b^	19	28.8	87	46.3	.013^c^	19	28.8	64	48.5	.008^c^
Diarrhea	7	36.8	37	42.5	.649^c^	7	36.8	28	43.8	.592^c^
Neurological symptoms	1	5.3	3	3.4	.552^d^	1	5.3	3	4.7	>.999^d^
Elevation of liver-enzyme levels	5	26.3	23	26.4	.991^c^	5	26.3	20	31.3	.681^c^
Renal insufficiency	2	10.5	9	10.3	.981^c^	2	10.5	8	12.5	.816^c^
Electrolyte disturbances	0	0.0	1	1.1	>.999^d^	0	0.0	1	1.6	>.999^d^
Nausea, vomiting	2	10.5	29	33.3	.048^c^	2	10.5	19	29.7	.092^c^
Others	6	31.6	15	17.2	.155^c^	6	31.6	7	10.9	.030^c^
**Interval between start of COVID-19 treatment and ADR**					.050^e^					.016^e^
Median days, IQR	1	0–2	2	1–4		1	0–2	2	1–4	
Presence of drug–drug interactions	60	90.9%	174	92.6%	.670^c^	60	90.9	122	92.4	.712^c^
No. of drug–drug interactions					.163^e^					.285^e^
Median days, IQR	3	1–5.5	3.5	1–7		3.0	1–5.5	3.0	1–7	
**No. of drug–drug interactions directly related to COVID-19 treatment**					<.001^e^					<.001^e^
Median days, IQR	1	1–3	3	1–5		1.0	1–3	3.0	1–5	

Note. SARS-CoV-2, severe acute respiratory coronavirus virus 2; RT-PCR, reverse transcriptase–polymerase chain reaction; COVID-19, coronavirus disease 2019; ADR, adverse drug reactions; IQR, interquartile range.

a Percentage unless otherwise specified.

b Some patients had >1 adverse drug reaction simultaneously.

c Data presented as no. and %, compared by χ^2^ test.

d Data presented as no. and % compared using the Fisher exact test.

e Data presented as median and interquartile range, compared using the Mann-Whitney *U* test.

We detected no evidence of a significant difference in the overall prevalence of drug–drug interactions (*P* > .05) in the 2 groups. However, when considering interactions related to COVID-19 treatment drugs, the PCR-positive group had significantly more interactions (median 3 vs 1; *P* < .001). We detected no significant differences between groups regarding QT interval changes (*P* > .05) (Table 4). However, there was a significant difference between the QT interval values of the ECGs obtained at baseline and those obtained during treatment (*P* = .035). Of 70 patients who underwent baseline and treatment ECGs, 62 patients had normal QT intervals at baseline and 12 (19.4%) developed QT prolongation during treatment. Of the 8 patients with a prolonged QT at baseline, 5 (62.5%) remained prolonged and 3 (37.5%) normalized during treatment. Of the 17 patients who had a difference between baseline ECG and the ECG during treatment, 14 received combination therapy with hydroxychloroquine and azithromycin. Among them, an average of 2.71 prescription drugs with known risk for QT prolongation were prescribed, and 16 of these prescriptions showed the presence of drug interactions.

**Table 4.** Electrocardiogram Data by Group

Variable	Full Cohort				*P* Value	Propensity Score–Matched Cohort				*P* Value
	Negative SARS-CoV-2 RT-PCR (n = 66)		Positive SARS-CoV-2 RT-PCR (n = 188)			Negative SARS-CoV-2 RT-PCR (n = 66)		Positive SARS-CoV-2 RT-PCR (n = 132)		
	No.	%^a^	No.	%^a^		No.	%^a^	No.	%^a^	
Baseline ECG performed	31	47.0	92	48.9		31	47.0	63	47.7	
QT prolongation on baseline ECG^b^	3	9.7	11	12.0	.730^c^	3	9.7	5	7.9	.776^c^
**QT interval value at baseline ECG**					.921^d^					.840^d^
Mean, SD	419.00	29.50	419.65	32.48		419.00	29.50	420.35	31.96	
Minimum maximum	366.0	494.0	358.0	520.0		366.0	494.0	358.0	520.0	
Median and interquartile range	413.0	402–436	416.0	393.5–440		413.0	402–436	420.0	398–443	
ECG during treatment	18	27.3	107	56.9		18	27.3	76	57.6	
QT prolongation during treatment^b^	3	16.7	19	17.8	.911^c^	3	16.7	14	18.4	.862^c^
**QT interval value during treatment**					.992^e^					.863^e^
Mean, SD	427.89	29.48	429.49	38.12		427.89	29.48	431.26	39.94	
Minimum maximum	387.0	488.0	355.0	540.0		387.0	488.0	355.0	540.0	
Median and interquartile range	423.5	402–448	425.0	401–448		423.5	402–448	427.5	403–450	

Note. SARS-CoV-2, severe acute respiratory coronavirus virus 2; RT-PCR, reverse transcriptase–polymerase chain reaction; ECG, electrocardiogram; baseline ECG, electrocardiogram prior to starting treatment for COVID-19; SD, standard deviation.

a Percentage unless otherwise specified.

b QTc > 470 ms for men and QTc >480 ms for women.

c χ^2^ test.

d Student *t* test.

e Mann-Whitney *U* test.

**Fig. 1.**
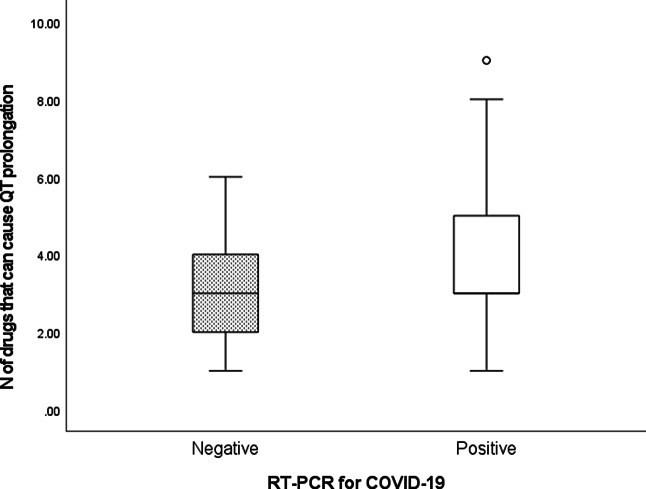
Number of drugs that can cause QT prolongation. Note. RT-PCR, reverse transcriptase–polymerase chain reaction.

Table 5 shows the utilization of drugs at risk of widening the QT interval. Our comparison included the number of drugs used as well as the risk classification for QT prolongation (ie, known, possible, or conditional). Patients with a positive PCR result for SARS-CoV-2 were significantly more likely to receive agents that cause QT prolongation at any risk classification level (*P* = .029), as well as a higher proportion of agents with known risk (*P* < .001) (Fig. 1).

**Table 5.** Number of Drugs Causing QT Prolongation by Group

Variable	Full Cohort				*P* Value	Propensity Score–Matched Cohort				*P* Value
	Negative SARS-CoV-2 RT-PCR (n = 6)		Positive SARS-CoV-2 RT-PCR (n = 188)			Negative SARS-CoV-2 RT-PCR (n = 66)		Positive SARS-CoV-2 RT-PCR (n = 132)		
	No.		No.			No.		No.		
**No. of drugs that can cause QT prolongation**					.021^a^					.029^a^
Mean, SD	3	1	4	2		3.24	1.23	3.96	1.87	
Minimum, maximum	1	6	1	9		1.0	6.0	1.0	9.0	
Median and interquartile range	3	2–4	3	3–5		3.0	2–4	3.0	3–5	
**No. of drugs with a known risk of TdP**					<.001^a^					<.001^a^
Mean, SD	2	1	3	1		1.95	0.92	2.50	0.97	
Minimum, maximum	1	5	1	6		1.0	5.0	1.0	6.0	
Median and interquartile range	2	1–3	2	2–3		2.0	1–3	2.0	2–3	

Note. SARS-CoV-2, severe acute respiratory coronavirus virus 2; RT-PCR, reverse transcriptase–polymerase chain reaction; SD, standard deviation; TdP, Torsades de Pointes.

a Mann-Whitney *U* test.

By the univariate model, use of hydroxychloroquine plus azithromycin (OR, 2.133; *P* = .027), duration of treatment (OR, 1.080; *P* = .018), and the number of drugs posing risk for QT interval prolongation (OR, 1.225; *P* = .014) were associated with a greater chance of developing an ADR (Table 6).

**Table 6.** Univariate Analysis of Risk Factor for Adverse Drug Reactions

Variables	OR (95% CI)	*P* Value
Drug used		
Hydroxychloroquine + azithromycin	2.133 (1.090–.175)	*.027*
Hydroxychloroquine	2.314 (0.806–.642)	.119
Azithromycin	Reference	…
Treatment duration, d	1.080 (1.014–.151)	*.018*
**Drug interactions**		
Yes	2.328 (0.738–.342)	.149
No	Reference	…
Length of stay, days	1.006 (0.996–.016)	.244
No. of drugs that can cause QT prolongation	1.255 (1.048–.503)	*.014*

Note. OR, odds ratio; CI, confidence interval. *P* value in italic indicates statistical significance.

## Discussion

During the study period, several drugs were being used around the world as potential treatments for COVID-19, including hydroxychloroquine, chloroquine, azithromycin, tocilizumab, and antivirals, in an attempt to reduce the mortality caused by the disease. Subsequently, randomized clinical trials showed that these drugs are not effective in the treatment of COVID-19.^20–22^ Nonetheless, despite the evidence, some ineffective drugs continue to be used, and there is often a delay between the emergence of evidence and its application in the clinical setting. During this delay, patients may be harmed by ineffective therapies.

Importantly, our study demonstrates the risks of ADRs and drug–drug interactions in patients empirically treated for COVID-19 that were shown by PCR to not be infected. In our study, the most commonly used drug was azithromycin, which was used in 88.6% of PCR-positive patients and in 84.8% of PCR-negative patients, followed by hydroxychloroquine, which was used in 84.8% of PCR-positive patients and 51.5% of PCR-negative patients. The low utilization of lopinavir/ritonavir in our hospital was due to the shortage of this medicine at the beginning of the pandemic, and the antiviral umifenovir is not marketed in Brazil.

Almost half (48.5%) of the patients with a confirmed diagnosis of COVID-19 had some type of ADR. Considering the likelihood of underreporting, that number may actually be even higher. Patients with confirmed COVID-19 remained hospitalized longer, which may have contributed to the greater number of ADRs in this group; indeed, the chance of the patient developing an ADR will be greater with greater exposure to that drug. The chance of this being reported and documented is higher with longer hospital stay; therefore, a propensity score was performed to take this factor into account. We framed as a limitation the fact that it was not possible to match based on the length of stay, due to the insufficient number of unconfirmed patients with COVID-19 with a length of stay long enough for an adequate match.

Sun et al^23^ reported an ADR incidence rate of 37.8%; this Chinese study included mostly female patients, unlike our study, in which the majority of patients were male. The mean age in our study was 65 years and in the study by Sun et al, the mean age was 46 years. In the Chinese study, 28.6% of patients had some underlying disease; in our study, 85.6% of patients diagnosed with COVID-19 had some underlying disease, 49.2% of whom had heart disease. The age difference between the studies is considerable, and older patients, as in our study, have a higher number of comorbidities and tend to take a greater number of medications.^24^

Approximately 43.8% of the ADRs identified were diarrhea, followed by elevation of liver-enzyme levels (31.3%). Sun et al^23^ also recorded most ADRs as disorders of the gastrointestinal tract (23%), followed by liver enzyme changes (13.8%). In a study by Surapat et al^25^ in Thailand, diarrhea was also the most common adverse reaction, occurring in 9% of patients who used protease inhibitors (lopinavir/ritonavir and darunavir/ritonavir). Notably, however, 2%–50% of patients diagnosed with COVID-19 reported diarrhea,^26–29^ and this is a limitation of any study that seeks to identify ADRs. We used the Naranjo algorithm to determine causality between the ADR and the drug,^11^ and most ADRs in our study were classified as possible. The pharmacist plays an important role in identifying ADRs and seeking the probability of a causal relationship with the administered drugs. Pharmacists are needed to prevent unnecessary ADRs and to alert physicians about the potential for ADRs, ensuring greater safety. Most patients also received antimicrobials, which may also be related to some of the reported ADRs, but the proportions between the groups were similar. Moreover, 62 (93.9%) of 66 patients in the group negative for SARS-CoV-2 by PCR also received antimicrobials, as did 176 (93.6%) of 188 patients in the PCR-positive group.

Despite prolongation of the QT interval being a worrisome ADR that has been identified in large studies, it was uncommon in a Thai study^25^ and also in our study. It was possibly underdetected due to our small sample and because ECG monitoring is only performed regularly in intensive care units and in patients at high risk for developing QT prolongation. Another factor that may have affected our findings is the lack of a protocol to guide the request and frequency of monitoring through ECG, leaving this decision to the discretion of the physician. We emphasize the importance of the clinical pharmacist in analyzing medical prescriptions and identifying potential risks and in alerting the medical team to request exams more frequently to ensure safe therapies, especially those that are administered off label. However, even with this small sample size, a subanalysis among patients who presented a significant difference in the value of the QT interval during treatment showed that 14 of the 17 patients received the associated drugs, which reinforces the need for monitoring of the QT interval.

A study by the pharmacovigilance center in France characterized the ADRs reported with the off-label use of azithromycin, hydroxychloroquine, chloroquine, and lopinavir/ritonavir. Among the 131 reports, 120 were related to cardiac events.^30^ Of the 120 reports, 85.8% were associated with the use of hydroxychloroquine, and approximately half of these were treated concurrently with azithromycin (findings similar to ours), and there was an association with these 2 drugs in 55.1% of the cases. Of the 131 adverse events reported, 68.7% were prolonged QT intervals and 30.8% of patients in the reports also received other drugs that could cause prolonged QT intervals, including escitalopram, spiramycin, and levofloxacin.^30^

In our study, in addition to the prescription of azithromycin and/or hydroxychloroquine, which are drugs known to have a known risk for Torsades de Pointes (TdP),^12^ in the SARS-CoV-2 PCR-positive group other QT prolonging agents used were proton pump inhibitors (67.6%), ondansetron (29.8%), dexmedetomidine (17%), and quetiapine (14.9%). On average, 4 agents known to prolong QT were prescribed to PCR-positive patients and 3 such agents were prescribed to PCR-negative patients (*P* = .029). Also, an average of 3 agents with a known risk for TdP were prescribed to SARS-CoV-2 PCR-positive patients and 2 such agents were prescribed to PCR-negative patients (*P* < .001). This finding highlights the importance of monitoring patients through ECG and/or through the observation of clinical conditions that can facilitate or induce TdP, such as electrolyte disorders and drug–drug interactions. We believe that the capacity of this French center to identify adverse cardiac reactions was extremely systematic and properly structured where it was possible to identify these on a large scale, different from the low numbers identified in our study and in the Thai study.^25^

The risk of drug interactions is higher in the elderly population, those with multiple comorbidities, as well as in the critical care environment.^31^ Drug–drug interactions result from their pharmacokinetic and pharmacodynamic properties and, in patients with COVID-19, an inflammatory response can alter the pharmacokinetic behavior of drugs.^31,32^ This factor highlights the need for monitoring of care by a clinical pharmacist.

In our study, only 94 patients were monitored by ECG before the start of medications, a low proportion given the resources available at our institution. Among the monitored PCR-positive patients, 18.4% had QT interval prolongation during their COVID-19 treatment. We cannot confirm that this was solely due to drugs because other factors can contribute to QT prolongation: electrolyte disorders, acute coronary insufficiency, bradycardia, and bundle branch block, among others.^33^ Ideally, an ECG monitoring protocol should be instituted at the beginning and during such drug therapies.

Some studies have shown that the ADRs were significantly associated with length of stay,^23,24^ but our study did not show this relationship. However, the duration of drugs involved COVID-19 treatment, the number of drugs used that can cause QT prolongation, and the use of hydroxychloroquine plus azithromycin were associated with a greater risk of ADRs in our univariate analysis.

Monitoring of ADRs and drug–drug interactions can be done through clinical monitoring of signs and symptoms and by monitoring laboratory tests and diagnoses. However, such monitoring can be challenging because it is often not possible to ascertain whether the symptoms presented are due to the disease itself or any medication being used. The work of a multidisciplinary team contributes to patient safety, and the establishment of therapeutic protocols is important and contributes to effective and safe therapy, especially in the context of new diseases.
